# Workplace violence against hospital healthcare workers in China: a national WeChat-based survey

**DOI:** 10.1186/s12889-020-08708-3

**Published:** 2020-04-29

**Authors:** Yusheng Tian, Yuchen Yue, Jianjian Wang, Ting Luo, Yamin Li, Jiansong Zhou

**Affiliations:** 1 Clinical Nursing Teaching and Research Section, The Second Xiangya Hospital, Central South University, Changsha, 410011 China; 2 Department of Psychiatry & Mental Health Institute, The Second Xiangya Hospital, Central South University, Changsha, 410011 China; 3 XiangYa School of Nursing, Central South University, Changsha, 410013 China; 4 Department of Psychiatry, CIUSSSE-CHUS - Hôtel-Dieu, Université de Sherbrooke, 580, rue Bowen Sud, Sherbrooke, Québec Canada

**Keywords:** Healthcare workers, Workplace violence, Survey

## Abstract

**Background:**

Workplace violence (WPV) is a serious issue for healthcare workers and leads to many negative consequences. Several studies have reported on the prevalence of WPV in China, which ranges from 42.2 to 83.3%. However, little information is available regarding the correlates of WPV among healthcare workers and the differences across the different levels of hospitals in China. This study aimed to explore the correlates of WPV and career satisfaction among healthcare workers in China.

**Methods:**

A self-designed WeChat-based questionnaire was used that included demographic and occupational factors. The Chinese version of the Workplace Violence Scale was used to measure WPV. Career satisfaction was assessed using two questions about career choices. Descriptive analyses, chi-square tests and multivariate logistic regressions were used.

**Results:**

A total of 3706 participants (2750 nurses and 956 doctors) responded to the survey. Among the 3684 valid questionnaires, 2078 (56.4%) reported at least one type of WPV in the last year. Multivariate logistic regressions revealed that male sex, shift work, bachelor’s degree education, a senior professional title, working more than 50 h per week and working in secondary-level hospitals were risk factors associated with WPV. Healthcare workers who had experienced higher levels of WPV were less likely to be satisfied with their careers.

**Conclusions:**

WPV remains a special concern for the Chinese healthcare system. Interventions to reduce WPV should be implemented by health authorities to create a zero-violence practice environment.

## Background

According to the National Institute for Occupational Safety and Health (NIOSH), workplace violence (WPV) is defined as acts directed towards persons at work or on duty [1]. WPV can be classified as physical assault (PA, physical contact, such as pushing, biting, beating, spitting), emotional abuse (EA, mistreatment through words, such as cursing, disrespect and disparagement), threats (T, use of verbal, written or physical force resulting in fear of negative consequences), verbal sexual harassment (VSH, unwelcome remarks or comments of a sexual nature), and sexual abuse (SA, unwanted touching or other sexual behaviours).

Aggression and violence toward healthcare workers constitute a global public health issue. Healthcare workers are vulnerable to WPV [2] as they are on the frontline of the healthcare system and have the closest contact with patients and their relatives [3]. Several studies have shown that WPV affects the physical and mental health of healthcare workers [4–8], resulting in reduced job satisfaction and performance [9, 10] and high rates of employee turnover and burnout [11–13], which ultimately leads to negative outcomes for patients [14, 15]. Furthermore, the treatment and indemnity for employees who report injuries in WPV are costly [16]. Many studies have examined the prevalence of WPV against healthcare workers [17–20]. In Australia, a study showed that verbal abuse (71%) was more common than physical violence (29%) [18]. In America, Pompeii et al. [21] reported that the 12-month prevalence of WPV among healthcare workers was 39.0%. In a repeated survey conducted by Johansen et al. [22] in Norway, no significant differences were found in the prevalence of self-reported threats (50.6% vs. 52.6%) or real acts of violence (23.9% vs. 25.3%) when comparing 2014 with 1993. Noorana et al. [23] reported that the prevalence of physical violence and non-physical violence for Indonesian emergency nurses was 10 and 54.6%, respectively. In a study conducted among nurses in South Korea [24], 74.3% of the respondents had experienced at least one type of verbal abuse during the past 3 months.

In mainland China, there were 6.2 million doctors and 3.8 million nurses in total according to the 2018 national health yearbook [25]. Several studies have reported the prevalence and correlates of WPV against healthcare workers in China [26–29], with the prevalence ranging from 42.2 to 83.3% [19, 30]. Male healthcare workers reported more WPV than females [3], inexperienced and graduate-level nurses were more vulnerable to WPV [11], and shift workers in the healthcare sector were at higher risk of WPV compared to non-shift workers [31, 32]. However, most studies have focused solely on township- or tertiary-level hospitals or on specialty healthcare groups. Little research has compared the prevalence of and factors associated with different types of WPV among doctors and nurses across the three levels of hospitals in China. Therefore, the objective of this research was to explore the frequency distribution of different types of WPV by demographic and occupational characteristics, to identify the associated factors for different types of WPV, and to investigate the impact of WPV on career satisfaction.

## Methods

### Participants and procedure

Convenience sampling was used to collect data. From January 10th to February 5th, 2019, this survey was conducted using a self-designed anonymous WeChat-based questionnaire. First, 40 nurses and doctors were selected as “original deliverers” from our university hospital. A link to our questionnaire survey was sent to them via social media (WeChat). The introduction of the study was shown on the first page of the questionnaire and participants chose “agree” to continue the survey or “disagree” to quit. Completing the survey questionnaire implied consent to join the study. Then, the colleagues or classmates of “the original deliverers” were invited to participate in the online survey (we encouraged the transfer of questionnaires among them, but no compensation was given). The questionnaire links were also distributed among the respondents’ friends and the WeChat groups. Subsequently, the size of the sample increased. The target group consisted of China’s front-line medical staff across different departments in the hospitals.

### Measures

The survey included sociodemographic information, such as gender, age, marital status, level of education, and occupational questions, including the level of hospital care, work department, profession, professional title, work schedule (shift or non-shift), weekly working hours and years of experience. Respondents also answered two questions regarding career choice: “Knowing all the risks, would you still have chosen the medical profession?” and “Would you want your child to become a healthcare worker?”

The frequency of WPV against Chinese healthcare workers in the previous 12 months was measured using the Chinese version of the Workplace Violence Scale (WVS), a scale with proven good reliability and validity (the Cronbach’s coefficient was 0.92) among healthcare workers in China [33]. The scale was composed of five dimensions, including PA, EA, T, VSH and SA. The score for each item ranged from 0 to 3, reflecting the frequency of the respondents’ exposure to WPV (0 = zero times, 1 = 1 time, 2 = 2 or 3 times, 3 = more than 3 times) in the past year. The total score was the sum of grades from each item, ranging from 0 to 15. The level of violence was divided into four categories according to the grades (none = 0, low = 1–5, intermediate = 6–10, high = 11–15). The survey provided specific definitions of each type of violence. Details about the questionnaire in this study are presented in the Additional file 1.

### Statistical analyses

The response to the question of whether the respondents suffered any type of WPV was coded as a dichotomous response (yes/no) in this study. The distribution of demographic and occupational data and the rates of five types of WPV were reported as numbers and percentages. The factors associated with each type of WPV at a priori specified *P*-value of 0.1 in the chi-square test were included in subsequent multivariate logistic regression analyses to identify significant predictors for the outcome variables, the five types of WPV. The association between career choice and the level of WPV was evaluated using the chi-square test. All analyses were performed using IBM software SPSS V.21.0 for Windows.

## Results

### Characteristics of the participants

A total of 3706 healthcare workers from all provinces in mainland China responded to the questionnaire, 22 of whom were excluded because of incomplete data. Among the 3684 respondents (74.6% nurses and 25.4% physicians), 65.8% were married, 34.2% were single, 84.9% were female, 69.0% worked in tertiary-level hospitals, 61.6% held a primary professional title and 73.3% were shift workers. The respondents were aged 18 to 72, and 50% of them were below the age of 30, with a mean age of 31.6 ± 7.7 years. The distribution of participants by demographic and occupational characteristics is shown in Table 1.

**Table 1:** The distribution of five types of WPV by demographic and occupational characteristics

**Demographic variables**	***n*** **= 3684** **N (%)**	**PA**	**EA**	**T**	**VSH**	**SA**
		**N (%)**	**N (%)**	**N (%)**	**N (%)**	**N (%)**
**Gender**						
Male	556(15.1)	134(24.1)	308(55.4)	212(38.1)	114(20.5)	72(12.9)
Female	3128(84.9)	452(14.5)	1481(47.3)	783(25.0)	453(14.5)	225(7.2)
*P* value		**< 0.01**	**0.01**	**< 0.01**	**< 0.01**	**< 0.01**
**Age group (yr)**						
< 30	1780(48.3)	284(15.9)	864(48.5)	399(22.4)	256(14.4)	139(7.8)
30–39	1306(35.5)	203(15.5)	645(49.4)	396(30.3)	215(16.5)	108(8.3)
≥ 40	598(16.2)	99(16.5)	280(46.8)	200(33.4)	126(21.1)	50(8.4)
*P* value		0.85	0.58	**< 0.01**	**< 0.01**	0.86
**Marital status**						
Married	2423(65.8)	365(15.1)	1150(47.5)	714(29.5)	395(16.3)	186(7.7)
Unmarried	1261(34.2)	221(17.5)	639(50.7)	281(22.3)	202(16.0)	111(8.8)
*P* value		0.58	0.07	**< 0.01**	0.83	0.25
**Education level**						
Master’s degree or above	437(11.9)	47(10.8)	185(42.3)	115(26.3)	68(15.6)	21(4.8)
Bachelor’s degree	2461(66.8)	434(17.6)	1235(50.2)	692(28.1)	416(16.9)	214(8.7)
Associate’s degree or below	786(21.3)	105(13.4)	369(46.9)	188(23.9)	113(14.4)	62(7.9)
*P* value		**< 0.01**	**< 0.01**	0.07	0.23	**0.02**
**Occupational variables**	***n*** **= 3684** **N (%)**	**PA**	**EA**	**T**	**VSH**	**SA**
		**N (%)**	**N (%)**	**N (%)**	**N (%)**	**N (%)**
**Level of hospital**						
Tertiary hospital	2541(69.0)	387(15.2)	1208(47.5)	683(26.9)	407(16.0)	190(7.5)
Secondary hospital	987(26.8)	190(19.3)	535(54.2)	295(29.9)	174(17.3)	99(10.0)
Primary/community hospital	156(4.2)	9(5.8)	46(29.5)	17(10.9)	16(10.3)	8(5.2)
*P* value		**< 0.01**	**< 0.01**	**< 0.01**	0.06	**0.02**
**Work department**						
Mental health	444(12.1)	216(48.6)	284(63.9)	196(44.1)	134(30.2)	92(20.7)
Intensive Care Unit	236(6.4)	69(29.2)	104(44.1)	58(24.6)	25(10.6)	18(7.6)
Emergency and Outpatient	308(8.4)	56(18.1)	197(63.9)	127(41.2)	71(23.1)	31(10.1)
Paediatric	165(4.5)	15(9.1)	90(54.5)	52(31.5)	27(16.4)	4(2.4)
Gynaecology and Obstetrics	217(5.9)	18(8.3)	91(41.9)	47(21.7)	27(12.4)	9(4.1)
Internal Medicine	969(26.3)	95(9.8)	488(50.4)	246(25.4)	128(13.2)	69(7.1)
Surgical Department	743(20.2)	79(10.6)	372(50.1)	177(23.8)	120(16.2)	49(6.6)
Operating Room	134(3.6)	6(4.5)	22(16.4)	17(12.7)	14(10.4)	3(2.2)
Diagnosis and Subsidiary	326(8.8)	20(6.1)	104(31.9)	55(16.9)	41(12.6)	18(5.5)
General	142(3.9)	12(8.4)	40(28.1)	20(14.1)	10(7.0)	4(2.8)
*P* value		**< 0.01**	**< 0.01**	**< 0.01**	**< 0.01**	**< 0.01**
**Profession**						
Nurse	2750(74.6)	437(15.8)	1324(48.1)	711(25.9)	419(15.2)	224(8.1)
Physician	934(25.4)	149(15.9)	465(49.8)	284(30.4)	178(19.1)	73(7.8)
*P* value		0.96	0.41	**< 0.01**	**< 0.01**	0.78
**Professional title**						
Senior	394(10.7)	71(18.0)	205(52.0)	146(37.1)	104(26.4)	38(9.6)
Intermediate	1021(27.7)	158(15.5)	492(48.2)	318(31.1)	166(16.3)	76(7.4)
Primary	2269(61.6)	357(15.7)	1092(48.1)	531(23.4)	327(14.4)	183(8.1)
*P* value		0.47	0.35	**< 0.01**	**< 0.01**	0.39
**Work schedule**						
Shift	2700(73.3)	478(17.7)	1416(52.4)	751(27.8)	459(17.0)	250(9.3)
Non-shift	984(26.7)	108(10.9)	373(37.9)	244(24.8)	138(14.0)	47(4.8)
*P* value		**< 0.01**	**< 0.01**	0.07	**0.03**	**< 0.01**
**Weekly working hours**						
> 50h	532(14.4)	94(17.7)	313(58.8)	169(31.8)	115(21.6)	43(8.1)
40-50h	2140(58.1)	315(14.7)	1028(48.0)	554(25.9)	315(14.7)	167(7.8)
< 40h	1012(27.5)	177(17.5)	448(44.4)	272(26.9)	167(16.5)	87(8.6)
P value		0.07	**< 0.01**	**0.02**	**< 0.01**	0.75
**Years of experience**						
< 5	1383(37.5)	223(16.1)	659(47.7)	296(21.4)	203(14.7)	112(8.1)
6–10	1054(28.6)	149(14.1)	525(49.8)	290(27.5)	152(14.4)	84(7.9)
11–20	766(20.8)	136(17.7)	390(50.9)	258(33.7)	150(19.6)	68(8.9)
> 20	482(13.1)	78(16.2)	215(44.6)	151(31.3)	92(19.1)	33(6.8)
*P* value		0.21	0.12	**< 0.01**	**< 0.01**	0.65

### The prevalence and characteristics of WPV

The rate of WPV among healthcare workers was 56.4% (2079/3684). EA had the highest rate (48.6%), followed by T (27.0%), VSH (16.2%), PA (15.9%), and SA (8.1%). The five types of WPV showed significant differences in their one-year prevalence by demographic and occupational characteristics (Table 1). Male healthcare workers had a higher prevalence of PA (24.1% vs. 14.5%, ***χ***^2^ = 38.9, *p* < 0.01), EA (55.4% vs. 47.3%, ***χ***^2^ = 12.2, *p* = 0.01), T (38.1% vs. 25.0%, ***χ***^2^ = 41.1, *p* < 0.01), VSH (20.5% vs. 14.5%, ***χ***^2^ = 45.3, *p* < 0.01) and SA (12.9% vs. 7.2%, ***χ***^2^ = 21.1, *p* < 0.01) than their female colleagues. Bachelor’s degree holders had the highest prevalence of PA (17.6% ***χ***^2^ = 17.9, *p* < 0.01), EA (50.2% ***χ***^2^ = 10.2, *p* < 0.01) and SA (8.7%***χ***^2^ = 7.6, *p* < 0.01) compared to the other groups classified by the level of education. Among the three levels of hospital care, medical staff in secondary hospitals had the highest prevalence of all five types of WPV (PA, EA, T, VSH and SA were 19.3, 54.2, 29.9, 17.3 and 10.0%, respectively). Mental health professionals were most vulnerable to all five types of violence (PA, EA, T, VSH, and SA were 48.6, 63.9, 44.1, 30.2 and 20.7%, respectively), followed by healthcare workers in emergency departments, in outpatient clinics and finally in paediatric departments. Shift workers appeared to be more vulnerable to PA (17.7% vs. 10.9%, ***χ***^2^ = 24.4, *p* < 0.01), EA (52.4% vs. 37.9%, ***χ***^2^ = 61.0, *p* < 0.01), VSH (17.0% vs. 14.0%, ***χ***^2^ = 4.7, *p* = 0.03) and SA (9.3% vs. 4.8%, ***χ***^2^ = 19.6, *p* < 0.01) than non-shift workers. Healthcare workers working more than 50 hours per week were more vulnerable to EA (58.8% ***χ***^2^ = 30.2, *p* < 0.01), T (31.8% ***χ***^2^ = 7.5, *p* = 0.02) and VSH (21.6% ***χ***^2^ = 15.0, *p* < 0.01).

### WPV levels and career choice

The correlation between WPV levels and career choice is shown in Table 2. A total of 880 (23.9%) of the respondents reported that they still would have chosen the medical profession even if they had been more aware of the risks, and 90.7% (3341/3684) indicated that they would not support their children becoming healthcare workers. Healthcare workers who had experienced higher levels of WPV were less likely (*P* < 0.01) to say “yes” when asked the above two questions. A total of 549 (14.9%) of the healthcare workers who had experienced high levels of WPV in the previous year reported that they still would choose a career in health care, while only 103 (2.8%) would support their children becoming healthcare workers.

**Table 2:** Association of career choice and the level of WPV

WPV level	N (%) (***n*** = 3684)	Still would have chosen a career in health care (%)		Would support children becoming healthcare workers (%)	
		Yes (23.9)	No (63.1)	Yes (9.3)	No (90.7)
**High**	71(1.9)	14.9	85.1	2.8	97.2
**Intermediate**	322(8.7)	15.8	84.2	5.9	94.1
**Low**	1686(45.8)	19.3	80.7	6.8	93.2
**None**	1605(43.6)	30.6	69.4	13.0	87.0
***P***		**<0.01**		**<0.01**	

### Associated factors for WPV

Table 3 shows the results of the multivariate logistic regression analyses on the associated factors for the five types of WPV. Male sex and shift work were two significant factors associated with all five types of WPV (*p* < 0.01). Bachelor’s degree education emerged as a significant factor associated with PA (*p* < 0.01), EA (*p* < 0.01) and SA (*p* < 0.01). The strongest correlate for PA (*p* < 0.01), EA (*p* < 0.01) and T (*p* < 0.01) was working in secondary hospitals, with odds ratios of 3.6 (95% CI: 1.79–7.31), 2.6 (95% CI: 1.76–3.75) and 3.3 (95% CI: 1.91–5.54), respectively. Holding a senior professional title was a factor associated with T (*p* = 0.01) and VSH (*p* = 0.01), and working more than 50 h per week [OR = 1.64 (95% CI: 1.32–2.05)] was a factor associated with EA (*p* < 0.01).

**Table 3:** Multivariate logistic regression for the association between demographic and occupational factors and five types of WPV

Risk factors	PA	EA	T	VSH	SA
	OR (95% CI)	OR (95% CI)	OR (95% CI)	OR (95% CI)	OR (95% CI)
**Male**	1.94(1.55–2.42)	1.31(1.09–1.59)	1.80(1.48–2.19)	1.74(1.39–2.18)	1.97(1.47–2.63)
**Shift work**	1.60(1.28–2.01)	1.65(1.41–1.92)	1.37(1.13–1.66)	1.48(1.18–1.86)	1.91(1.38–2.65)
**Education level**					
Master’s degree and above	Ref	Ref			Ref
Associate’s degree and below	1.35(0.92–1.99)	1.25(0.97–1.61)			1.76(1.03–3.00)
Bachelor’s degree	1.79(1.29–2.48)	1.39(1.12–1.72)			1.88(1.18–3.01)
**Level of hospital**					
Primary	Ref	Ref	Ref		
Tertiary	2.71(1.35–5.53)	2.01(1.39–2.91)	2.75(1.63–4.64)		
Secondary	3.62(1.79–7.31)	2.57(1.76–3.75)	3.25(1.91–5.54)		
**Professional title**					
Primary			Ref	Ref	
Intermediate			1.19(0.95–1.50)	1.24(1.00–1.53)	
Senior			1.54(1.09–2.16)	2.34(1.71–3.09)	
**Working hours/week**					
< 40h		Ref			
40-50h		1.16 (0.99–1.35)			
> 50h		1.64 (1.32–2.05)			

## Discussion

This study explored the correlates of WPV against Chinese healthcare workers and investigated the correlation between WPV and career satisfaction. The reported rate of WPV among healthcare workers was 56.4%, and the rate of EA was the highest (48.6%). Male sex, shift work, holding a bachelor’s degree, holding a senior professional title, working more than 50 h per week and working in secondary-level hospitals were independent factors associated with WPV. Healthcare workers who had experienced higher levels of WPV were less likely to be satisfied with their careers.

### Prevalence of WPV

This study showed that the overall prevalence of WPV among healthcare workers in mainland China (56.4%) was higher than Hong Kong’s WPV prevalence of 44.6% reported in a recent (2017) cross-sectional study of 850 healthcare workers [31] and lower than the overall national prevalence of 62.2% reported in a recent meta-analysis of 44 observational surveys [34]. The prevalence was also much lower than the prevalence of 76% reported in a national survey conducted among emergency nurses in Italy [35] and 67% among nurses and midwives in Australia [36].

### Occupational factors and WPV

Our results showed that the rate of WPV was higher in male healthcare workers than in females. This finding is consistent with those reported by previous studies [31, 32]. We found that work schedule (shift or non-shift work), working time and professional title also influenced the odds of exposure to WPV. These results are also consistent with findings from previous studies [32, 37]. Several reasons could explain the effects of shift work on the odds of experiencing the five types of WPV, including the shortage of staff on night shifts, staff exhaustion [38], and the consequent effect on patient satisfaction. In China, a normal hospital ward usually has only one or two nurses with one doctor on night shift, and they must take care of more than 40 patients. Healthcare workers working more than 50 h per week were found to be 1.64 times more likely to experience EA compared to those working less than 40 h per week. One possible explanation for this finding could be the increase in patient contact associated with longer weekly working hours, which increases the likelihood of encountering WPV. Healthcare workers with a senior professional title reported more T and VSH than those with primary titles. In China’s 3-tier responsibility system for doctors and nurses, a senior professional title usually brings greater responsibility and a heavier workload. According to Wu et al. [34], many senior doctors must see more than 100 outpatients on a given day, which may affect their health and the quality of their services. In addition, studies have revealed that patients tend to have higher expectations of physicians who hold senior professional titles [39], and unmet expectations constitute a major risk for assaults [40].

### Hospital level and WPV

Our findings showed a significant correlation between the level of hospital care and the risk of WPV. Healthcare workers in secondary-level hospitals had a higher risk of all five types of WPV than those working in primary- and tertiary-level hospitals. Previous studies have identified several factors that could contribute to this finding. First, the number of both outpatients and inpatients in China’s secondary-level hospitals is increasing due to hierarchical diagnosis and treatment reform, which aims to manage simple diseases at the township level and in secondary-level hospitals [41]. Furthermore, in China, secondary-level hospitals suffer greater staff shortages than tertiary-level hospitals [42]. The increasing patient load and staff shortage lead to higher workload and higher risk of burnout for healthcare workers in secondary-level hospitals, which may subsequently result in negative outcomes for patients [43]. In addition, secondary-level hospitals are less advanced relative to the staff’s level of education and the sophistication of medical equipment than tertiary-level hospitals [42, 44], which causes discrepancies in the quality of medical services and may lead to more medical disputes as well as a higher level of dissatisfaction among patients and their family members. The majority of workplace aggression was performed by patients who suffered negative clinical outcomes and by their dissatisfied family members [40, 45]. However, healthcare workers in primary-level hospitals suffered less WPV than those in secondary- and tertiary-level hospitals. A possible explanation for this finding is that primary-level hospitals provide fundamental medical services in relatively smaller areas where staff tend to know their clients better and tend to develop better patient-physician relationships, making them less likely to suffer from WPV.

### Career satisfaction and WPV

This study showed that the higher the level of violence, the less likely healthcare workers were to answer “yes” to the two questions regarding career choice. WPV may adversely affect the physical and mental health of healthcare workers [4–8], resulting in decreased job satisfaction [46], poorer quality of life [9] and an increased risk of staff burnout and employee turnover [47]. This situation is worsened by the lack of condemnation from the public towards the perpetrators following the news of doctors murdered due to WPV [48]. These negative effects and citizens’ attitudes towards hospital WPV may have decreased the victims’ confidence in the healthcare system, leading them to say “no” when asked the two questions.

### Limitations

This study has several limitations. First, the data were collected retrospectively. This method depends on the respondents’ ability to recall events that occurred in the previous 12 months, potentially resulting in recall bias. Second, this study was a national online cross-sectional survey, and although 3706 doctors and nurses from all provinces in mainland China responded, the sample was small and disproportionately distributed compared to the large number of Chinese health care workers. Therefore, the sample has limited representativeness, and the prevalence measures in this study cannot fully represent the current status of WPV among Chinese healthcare workers. Nevertheless, the significant risk factors of WPV found in this study have the potential to mitigate WPV towards healthcare workers in China.

## Conclusions

WPV remains a special concern for the Chinese healthcare system. The prevalence of WPV against healthcare workers in China identified in this study was high, and many associated factors were found. Healthcare workers in secondary-level hospitals raised more concerns as they were more vulnerable to WPV than those in primary and tertiary-level hospitals. Healthcare staff who had experienced higher levels of WPV were less likely to be satisfied with their careers. Interventions to reduce WPV should be implemented by health authorities to create a zero-violence practice environment.

## Supplementary information

**Additional file 1.** Details of the questionnaire in the study.

## Data Availability

The datasets used and/or analysed in the current study are available from the corresponding authors on reasonable request.

## Abbreviations

NIOSH: National Institute for Occupational Safety and Health
WPV: Workplace violence
PA: Physical assault
EA: Emotional abuse
T: Threat
VSH: Verbal sexual assault
SA: Sexual assault
