# Supplementary material for: Workplace violence against hospital healthcare workers in China: a national WeChat-based survey
Source: BMC Public Health. 2020 Apr 29;20:582. doi: 10.1186/s12889-020-08708-3 (PMC7189471; doi:10.1186/s12889-020-08708-3)
Supplement: Supplementary file 1 — Additional file 1. Details of the questionnaire in the study. [file 12889_2020_8708_MOESM1_ESM.doc]

**Additional file 1:** Details of the questionnaire in the study.

**1. Demographic information**

1. Your Gender

£male

£female

1. Your Age:
2. Your Marital Status

£married

£unmarried

1. Your Education level:

□ Master’s degree or above

£Bachelor’s degree

£Associate’s degree or below

**2. Professional information**

1. The level of the hospital you work in.

£tertiary

£secondary

£primary

1. Which department do you work in your hospital？
2. Are you a nurse or physician？

£nurse

£physician

1. Do you do shift work or not？

£shift

£non-shift

1. How many hours do you work in a week？ Hours
2. How many years have you been working as a healthcare provider？ Years
3. The professional title you hold now is？

£senior

£intermediate

£primary

1. **workplace violence scale**

***In the past year***, have you encountered the following types of violence from patients or patients’ relatives? Please choose the frequency listed besides after reading the definition of each type of violence.

| **5 types of violence** | **Times encountered in the past year** | | | |
| --- | --- | --- | --- | --- |
|  | None | 1time | 2-3times | ≥4times |
| 1.physical assault (pushing, biting, beating, spitting) |  |  |  |  |
| 2.emotional abuse(cursing, disrespect and disparagement words) |  |  |  |  |
| 3.threats(use of verbal, written or physical force resulting in fear of negative consequences) |  |  |  |  |
| 4.verbal sexual harassment (unwelcome remarks or comments of a sexual nature) |  |  |  |  |
| 5. sexual abuse ( unwanted touching or other sexual behaviors) |  |  |  |  |

1. **Career satisfaction questions**

If you have a chance, would you still have chosen the medical profession?

£Yes

£No

Would you want your child to become a healthcare worker?

£Yes

£No
